# Interference in melanoma CD248 function reduces vascular mimicry and metastasis

**DOI:** 10.1186/s12929-022-00882-3

**Published:** 2022-11-18

**Authors:** Cheng-Hsiang Kuo, Ya-Fang Wu, Bi-Ing Chang, Chao-Kai Hsu, Chao-Han Lai, Hua-Lin Wu

**Affiliations:** 1 International Center for Wound Repair and Regeneration, National Cheng Kung University, Tainan, Taiwan; 2 Department of Biochemistry and Molecular Biology, College of Medicine, National Cheng Kung University, No. 1, University Road, 701 Tainan, Taiwan; 3 Department of Dermatology, National Cheng Kung University Hospital, College of Medicine, National Cheng Kung University, Tainan, Taiwan; 4 Department of Surgery, National Cheng Kung University Hospital, College of Medicine, National Cheng Kung University, Tainan, Taiwan

**Keywords:** CD248, Vascular mimicry, Fibronectin, Metastasis, Melanoma

## Abstract

**Background:**

Tumor vascular mimicry is an emerging issue that affects patient survival while having no treatment at the current moment. Despite several factors implicated in vascular mimicry, little is known about stromal factors that modulate tumor microenvironment and shape malignant transformation. CD248, a type-I transmembrane protein dominantly expressed in stromal cells, mediates the interaction between cells and extracellular matrix proteins. CD248 protein expression is associated with the metastatic melanoma phenotype and promotes tumor progression in the stromal cells. This study aimed to explore the cell-autonomous effects of CD248 in melanoma vascular mimicry to aid cancer therapy development.

**Methods:**

Loss-of-function approaches in B16F10 melanoma cells were used to study the cell-autonomous effects of CD248 on cell adhesion, migration, proliferation, and vascular mimicry. A solid-phase binding assay was performed to identify the interaction between CD248 and fibronectin. Horizontal and vertical cell migration assays were performed to analyze cell migration activity, and cell-patterned network formation on Matrigel was used to evaluate vascular mimicry activity. Recombinant CD248 (rCD248) proteins were generated, and whether rCD248 interfered with melanoma CD248 functions was evaluated in vitro. An experimental lung metastasis mouse model was used to investigate the effect of rCD248 treatment in vivo.

**Results:**

CD248 protein expression in melanoma cells was increased by a fibroblast-conditioned medium. Knockdown of *CD248* expression significantly decreased cell adhesion to fibronectin, cell migration, and vascular mimicry in melanoma cells. The lectin domain of CD248 was directly involved in the interaction between CD248 and fibronectin. Furthermore, rCD248 proteins containing its lectin domain inhibited cell adhesion to fibronectin and slowed down cell migration and vascular mimicry. Treatment with rCD248 protein could reduce pulmonary tumor burden, accompanied by a reduction in vascular mimicry in mice with melanoma lung metastasis.

**Conclusion:**

CD248 expression in melanoma cells promotes malignant transformation by increasing the activity of cell adhesion, migration, and vascular mimicry, whereas rCD248 protein functions as a molecular decoy interfering with tumor-promoting effects of CD248 in melanoma cells.

## Background

Tumor metastasis, which drastically affects the survival rate of cancer patients, remains a significant obstacle in cancer treatment. Angiogenesis in solid tumors is a well-recognized hallmark of tumor progression. However, it has been demonstrated that anti-angiogenic therapy targeting vascular endothelial cells does not effectively inhibit cancer progression and potentially leads to aggressive tumor phenotypes, indicating that tumors may develop mechanisms to resist anti-angiogenic therapy [1]. It has been proposed that tumors might adapt three possible strategies to resist anti-angiogenic therapy: upregulation of alternative pathways (growth factors), endothelial cell-independent tumor perfusion [vascular mimicry (VM) or phenotype switch], and hijacking the pre-existing vessels (vessel co-option or pericytic mimicry) [1]. Notably, pericytic mimicry and VM are associated with the deterioration of patient survival rate and could be responsible for resistance to anti-angiogenic therapy [2]. Thus, understanding the molecular mechanism underlying malignant melanoma transformation would help develop a new therapeutic strategy.

Aggressive tumor cells might assume VM capacity to construct vessel-like conduits independent of endothelial cells to connect to blood circulation [3]. This tumor do-it-yourself channel was first discovered in human uveal melanoma and is histopathologically characterized by periodic acid-Schiff (PAS) stain-positive and CD31 stain-negative vessel-like structures, with blood cells enclosed in the tumor tissue Section [4]. VM has been observed in many tumor types, such as osteosarcoma, fibrosarcoma, glioblastoma, and carcinomas of the breast, colon, lung, pancreas, ovary, and liver, and is inversely correlated with patient survival [4–7]. Moreover, VM is a prominent behavior observed in highly invasive and metastatic melanoma cells but not in poorly invasive melanoma cells [8]. Although the mechanism of VM formation is still not completely understood, several important factors, including epithelial-mesenchymal transition, hypoxia, embryonic and stem cells, and vascular signaling pathways, have been implicated in VM in cancer [9–12]. Therefore, several molecular markers such as VE-cadherin and matrix metalloproteinases (MMPs) in VM have been identified [13]. In addition, the tumor microenvironment creates a niche that greatly affects tumor plasticity and the transition states associated with cancer functional heterogeneity [14]. Particularly, a recent study showed that fibroblasts can induce VM in lung cancer [15], suggesting that stromal factors play an essential role in the malignant transformation of cancer. Thus, cancer cells can adopt alternative pathways to acquire cellular plasticity and transform into an endothelial-like or vascular-like cell type to obtain a sufficient nutrient supply and extend to another level of cancer malignancy.

CD248, a member of the C-type lectin domain group XIV family of glycosylated transmembrane proteins, comprises six functional domains: the N-terminal lectin domain (D1), a Sushi domain (D2), three tandem-repeated epidermal growth factor (EGF)-like domain (D3), a mucin-like domain (D4), and a transmembrane domain (D5), which is followed by a cytoplasmic tail (D6). By binding to various extracellular matrix (ECM) molecules, CD248 has been found to participate in cell adhesion and cell migration [16]. CD248 is an oncofetal protein-like molecule that has upregulated levels in many pathological conditions, including tissue fibrosis and cancer [17], and is widely expressed in the fetus during embryogenesis. However, its expression is quickly downregulated after birth in mice and remarkably downregulated in adult tissues, except in the kidney [18]. Stromal CD248 promotes tumor growth and metastasis, partly through an increase in tumor cell extravasation and tumor environment construction [19, 20]. Elevated CD248 expression levels have been detected in several tumors, such as osteosarcoma and renal cell carcinoma [21, 22]. Moreover, CD248 expression has been detected in 85% of tumor microenvironment vasculature of metastatic melanoma; however, no expression was detected in the normal tissue samples, suggesting that CD248 may contribute to melanoma progression [23].

In this study, we investigated whether stromal factors could contribute to the malignant transformation of melanoma. We demonstrated the mechanism underlying the cellular autonomous effects of CD248 expression in aggressive phenotypes of melanoma cells associated with tumor metastatic behaviors. The role of CD248 in cell adhesion, proliferation, migration, and VM in melanoma was evaluated using a reductionist approach.

## Materials and methods

### Cell culture

B16F10 cells, HEK293 cells, and NIH3T3 cells were purchased from ATCC and maintained in Dulbecco’s Modified Eagle’s Medium (DMEM) containing 25 mM glucose and 10% fetal bovine serum (FBS). The NIH3T3 cell-conditioned medium (CM) was prepared by culturing NIH3T3 cells with the serum-free DMEM for 24 h. The CM was then centrifuged and stored at -80 ℃ until the use for the following experiment including chemotactic migration assay and VM assay. CD248 siRNA (siCd248) and scrambled siRNA control (siCtrl) were purchased from GE Dharmacon (siGENOME). The protein expression level was analyzed using a western blot assay with specific antibodies against CD248 (18160-1-AP, Proteintech), MMP9 (10375-2-AP, Proteintech), with GAPDH (sc-32,233, Santa Cruz), actin-β (HRP-60,008, Proteintech), or tubulin-α (HRP-66,031, Proteintech) as an internal control.

### Preparation of recombinant CD248 (rCD248) proteins

The human CD248 cDNA was used to construct rCD248 protein expression plasmids. The recombinant lectin domain of CD248 (rCD248D1) and the recombinant extracellular region of CD248 (rCD248D1-D4) were expressed using the pSecTag 2-A expression system in HEK293 cells. The recombinant EGF domain of CD248 (rCD248D3) was expressed using the pPICZα-A expression system in *Pichia pastoris*. All these rCD248 proteins comprise a c-Myc-tag and a His-tag at their C-terminus for detection and purification. Soluble rCD248 protein-containing medium was purified by using Q-*Sepharose* and nickel-chelating *Sepharose* chromatography.

### Cell migration assay

Chemotactic (vertical) migration assay and wound recovery (horizontal) migration assay were conducted to test cell migratory activity. The chemotactic migration assay was performed as previously described [24] using a Boyden chamber through a membrane with an 8-µm pore and CM as chemoattractant. The migrated cells were stained with Liu’s stain and enumerated 3 h after migration. The wound recovery migration assay was performed using a culture insert (ibidi) with confluent-grown cells in a culture medium with % FBS. The wound recovery ratio was measured as previously described [25]. For exogenous CD248 expression in CD248-null HEK293 cells, GFP or GFP-tagged human CD248 (CD248-GFP) expression plasmids were transfected into HEK293 cells. In the Boyden chamber migration assay with HEK293 cells, the membrane was coated with fibronectin and a medium containing 5% FBS was used as a chemoattractant. The HEK293 cells were allowed for migration for 4 h in the presence or absence of rCD248D1-4. To study the inhibitory effect of rCD248 proteins on chemotactic migration from the interaction with fibronectin, soluble fibronectin was pre-incubated with rCD248D1-4 proteins.

### Cell proliferation assay

The MTT assay was conducted to assess cell proliferation. The absorbance at 550 nm was measured after MTT treatment (Sigma).

### Cell adhesion assay

Cell adhesion assay was conducted as previously described with some modifications [26]. In brief, melanoma cells were allowed to adhere to fibronectin-coated or BSA-coated plates and incubated for 30 min. After the proper wash procedure, the adherent cells were stained using crystal violet, and the absorbance at 570 nm was measured. The level of focal adhesion kinase (FAK) phosphorylation was analyzed using a western blot assay with specific antibodies against Tyr576-phosphorylated FAK (sc-16,563, Santa Cruz), FAK (sc-558, Santa Cruz), and GAPDH, respectively.

### Solid-phase binding assay

The solid-phase binding assay was conducted to evaluate the interaction between CD248 and fibronectin as previously described with substantial modification [26]. Briefly, rCD248 proteins were added to a fibronectin-coated or BSA-coated plate, and the anti-c-Myc antibody was used to detect rCD248 proteins after appropriately washing. The absorbance at 450 nm was measured.

### VM assay—patterned network formation on Matrigel

Melanoma cells were seeded in µ-slide (ibidi) with Matrigel inside. To study whether the inhibitory effect of rCD248 proteins on VM from interaction with the fibronectin, soluble fibronectin was pre-incubated with rCD248D1-4 proteins. After the indicated period, the whole well was photographed, and the total tube length was measured.

### Immunohistochemistry

The general procedure for immunohistochemistry was followed as previously described with substantial modification [25]. The specific antibodies were used to detect the expression of CD248 (18160-1-AP, Proteintech), MMP9 (10375-2-AP, Proteintech), and CD31 (ab28364, Abcam) in lung tissue sections and counterstained with hematoxylin. To detect VM in tissue sections, a PAS stain was performed with a PAS staining kit (PSH-010, Baso).

### Experimental lung metastasis mouse model

The experimental mice were maintained in a specific pathogen-free animal facility at the Laboratory Animal Center of the National Cheng Kung University. The animal study was conducted following the experimental protocol (110085) approved by the Institutional Animal Care and Use Committee of National Cheng Kung University, and all procedures conformed to the NIH Guide for the Care and Use of Laboratory Animals. The mouse model of experimental metastasis was performed as previously with substantial modification [27]. In brief, eight-week-old male C57BL/6 mice were given an intravenous injection of rCD248D1-4 (20 µg) or PBS 1 h before an intravenous injection of 2 × 10^5^ B16F10 cells. On the other day, mice were given an intraperitoneal injection of rCD248D1-D4 (20 µg) or PBS once a day for 2 consecutive days. Fourteen days after tumor inoculation, mice were sacrificed. The lung surface tumor nodules were measured.

### Statistical analysis

Data are presented as the mean plus the standard error of the mean. Statistical analyses were performed using Graphpad Prism. Data were analyzed for normality and equal variance. Statistical analyses were performed with Student’s *t*-tests (unpaired and 2-tailed) or one-way ANOVA followed by Tukey’s multiple comparisons test when comparing more than two groups. *P* < 0.05 is considered statistical significance.

## Results

### Melanoma CD248 promotes cell adhesion onto fibronectin and migration

**Fig. 1:**
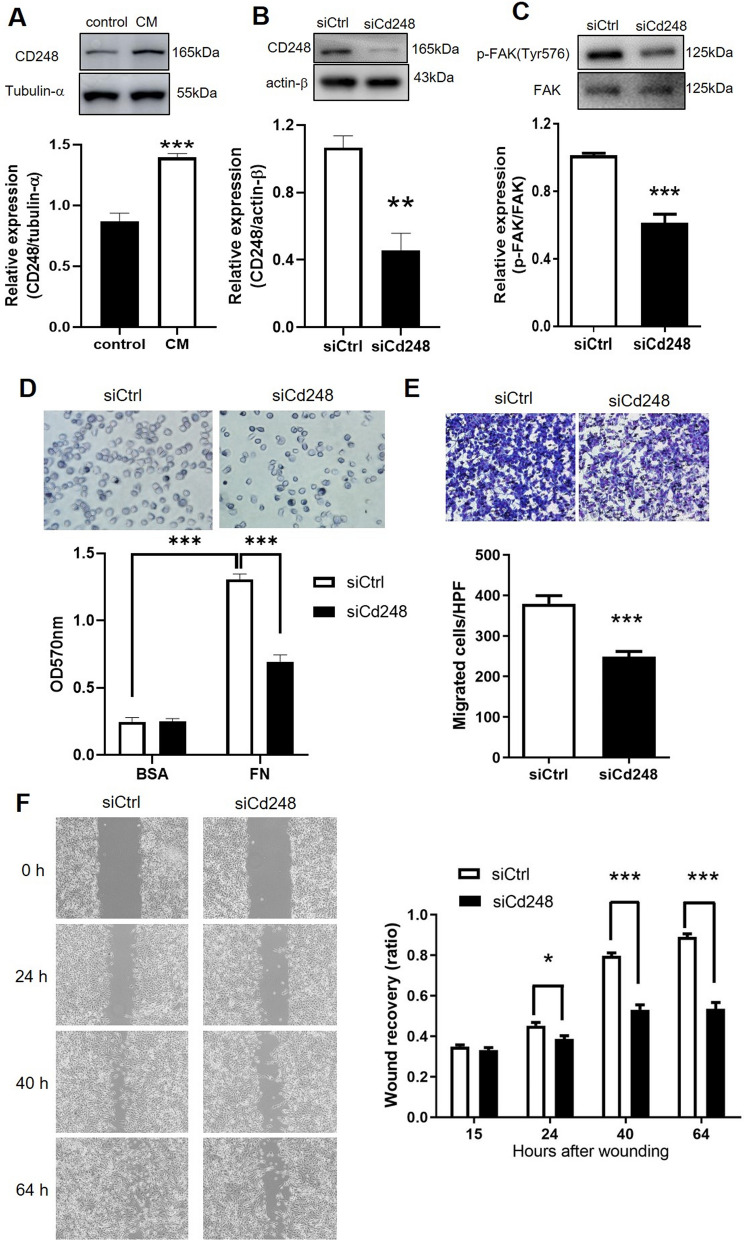
Melanoma CD248 promotes cell adhesion and migration. Mouse melanoma cells (B16F10 cells) transfected with siRNA against mouse Cd248 (siCd248) or with scrambled control siRNA (siCtrl) were subjected to cell adhesion and migration assays. **A** Western blot analysis of CD248 expression in B16F10 cells treated with the fibroblast-conditioned medium (CM) for 1 day and the statistical analysis thereof. N = 5. ***P < 0.001. **B** Western blot analysis of CD248 expression in B16F10 cells transfected with siCtrl or siCd248. N = 5. ***P < 0.001. The cells were subjected to **C**, **D** cell adhesion assay, **E** chemotactic migration assay, and **F** wound recovery assay. **C** Western blot analysis of FAK activation (phosphorylation of Tyr576 FAK, p-FAK(Tyr576) in cells after cell adhesion assay. N = 5. ***P < 0.001. **D** Representative images of cells 30 min after adhesion assay to fibronectin (FN)-coated multiple-well plate and the statistical analysis thereof. The level of cell adhesion was determined by measuring the number of adherent cells after staining with crystal violet followed by the measurement of OD570nm. N = 5. ***P < 0.001. **E** Representative images of migrated cells 3 h after chemotactic migration followed by Liu’s stain. Chemotaxis was induced by the CM. Statistical analysis of chemotaxis assay. N = 5. ***, *P* < 0.001. **F** Representative images of wound recovery assay and statistical analysis thereof. N = 8. *P < 0.05; ***P < 0.001

Fibronectin promotes melanoma proliferation and metastasis [28]. CD248 has been proposed to mediate cell-fibronectin interactions and migration in an exogenous cell model [16], yet the role of native CD248 in melanoma cell adhesion and migration remains to be investigated. We showed that CD248 was expressed in B16F10 cells, which was upregulated by a conditioned medium produced by fibroblasts (Fig. 1A). Knockdown of *CD248* in melanoma cells using a specific siRNA markedly reduced CD248 protein expression (Fig. 1B). Melanoma cells adhered to the fibronectin-coated surface in 30 min, but the adhesion and FAK activation were considerably reduced when melanoma CD248 expression was suppressed by siRNA (Fig. 1C, D). We investigated whether CD248 affects melanoma cell migration utilizing two different migration models. Chemotactic cell migration assay showed that cell migration toward CM was observed after 3 h (Fig. 1E); however, it was attenuated in siCd248-transfected cells (Fig. 1E). In the horizontal migration assay, the acellular space was almost entirely covered by relocated cells in siCntl-transfected cells but not in CD248-knockdown cells (Fig. 1F). Calculating the acellular area before and after migration revealed that reduced CD248 expression in melanoma cells retarded wound recovery (Fig. 1F). Thus, *CD248* knockdown in melanoma cells inhibited either vertical chemotaxis (Fig. 1E) or horizontal cell migration (Fig. 1 F). Taken together, these results indicate that melanoma CD248 plays a vital role in cell-fibronectin interactions and migration.

### Melanoma CD248 promotes VM but does not affect cell growth

**Fig. 2:**
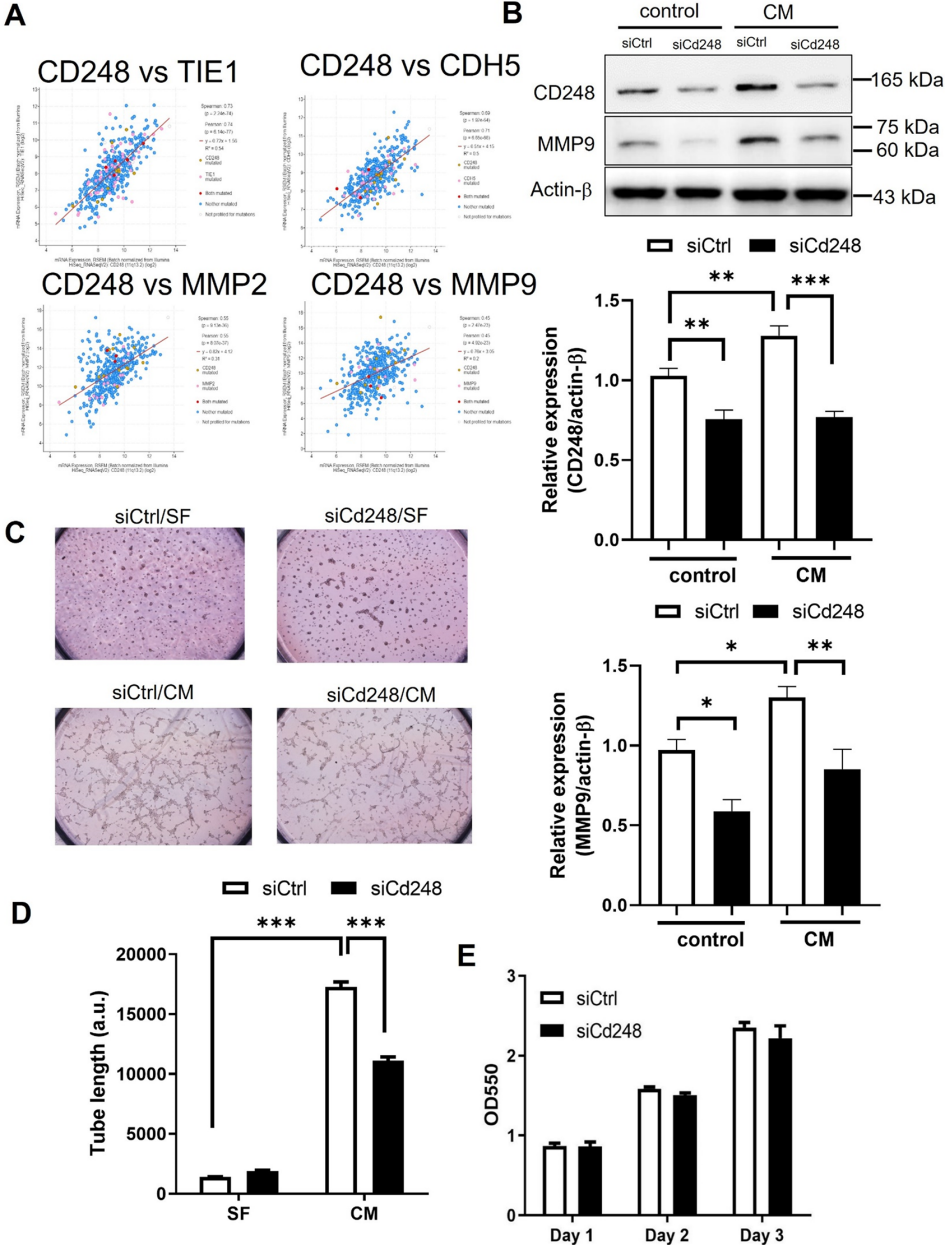
Melanoma CD248 promotes vascular mimicry but does not affect cell growth. **A** Analysis of the correlation of mRNA expression between CD248 and molecular markers for VM in the human melanoma tissues using the cBioPortal database. B16F10 cells transfected with siCtrl or siCd248 were subjected to **B** western blot assay, **C**, **D** VM assay (network formation on Matrigel), and **E** cell proliferation assay (MTT assay). **B** Western blot analysis of the relative expression of CD248 and MMP9 in cells treated with fibroblast-cultured conditioned medium (CM) with non-cell cultured medium (SF) as the control for 1 day. N = 6. *P < 0.05; **P < 0.01; ***P < 0.001. **C** Representative images of VM assay induced by CM with SF as control. **D** Statistical analysis of the total length of the network measured 6 h after seeding on Matrigel. N = 5. ***P < 0.001. **E** MTT assay. B16F10 cells with siCd248 or siCtrl were cultured for 1 to 3 days and followed by an MTT assay at indicated time point. N = 4

Aggressive melanoma phenotypes contribute to VM progression accompanied by the expression of molecular markers such as VE-cadherin and MMP2/9 [8, 13]. CD248 protein expression has been observed in 70% of melanoma specimens with vessel-like patterns [23]. Therefore, we investigated whether CD248 was involved in melanoma VM. By analyzing the correlation of the expression between CD248 and molecular markers of VM in melanoma tissues using the cBioPortal database (https://www.cbioportal.org/), several strong correlations such as TIE−1 (Spearman correlation ρ = 0.73, p = 2.24e−74; Pearson correlation ρ = 0.74, p = 6.14e−77), CDH5 (Spearman correlation ρ = 0.69, p = 1.92e−64; Pearson correlation ρ = 0.71, p = 6.65e−68), MMP2 (Spearman correlation ρ = 0.55, p = 9.13e−36; Pearson correlation ρ = 0.55, p = 8.07e−37), and MMP9 (Spearman correlation ρ = 0.45, p = 2.47e−23; Pearson correlation ρ = 0.45, p = 4.92e−23) were identified (Fig. 2A). Therefore, we analyzed MMP9 expression in B16F10 cells. The results showed that the MMP9 protein level was increased by CM while reduced when CD248 expression was inhibited by siRNA (Fig. 2B). In addition, melanoma cells formed a patterned network connection on Matrigel in response to CM (Fig. 2C), suggesting that melanoma VM activity could be induced by fibroblast CM. *CD248* knockdown significantly reduced VM formation in Matrigel (Fig. 2D). CD248 has been associated with cell proliferation in fibroblasts [25]. Thus, we speculated whether CD248 expression is associated with melanoma cell growth. The MTT assay using melanoma cells with siCd248 showed that melanoma CD248 expression level might not affect cell proliferation (Fig. 2E). These observations, together with other studies [16], suggest that CD248 may interact with ECM proteins, thereby promoting cell migration and VM.

### The lectin domain of CD248 binds to the N-terminal 70 kDa fragment of fibronectin

**Fig. 3:**
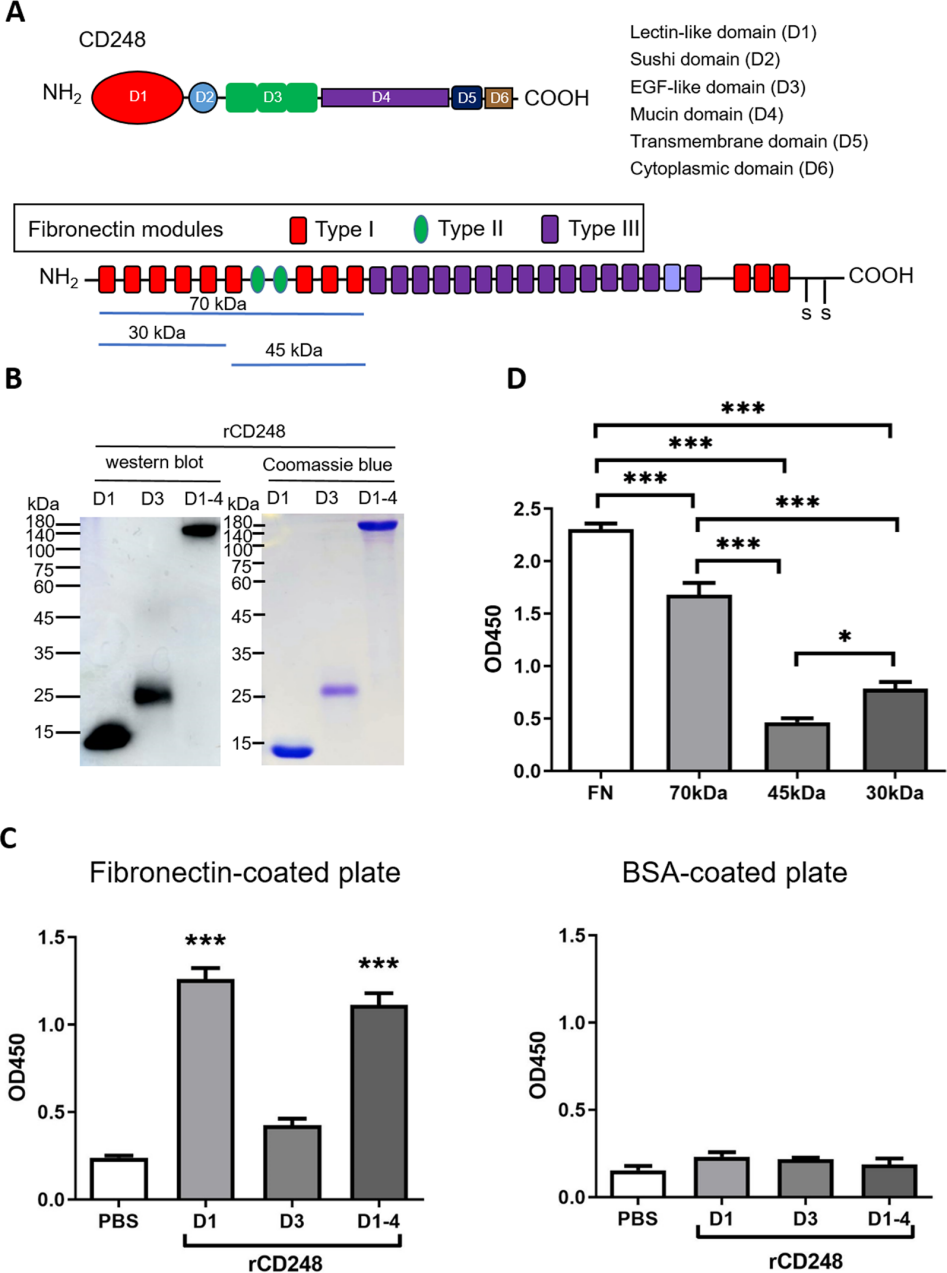
The lectin domain of CD248 and the N-terminal 70-kDa fragment of fibronectin mediate CD248-fibronectin interaction. **A** Schematic presentation of CD248 protein structure and fibronectin fragments used in this study. **B** Protein stain (Coomassie blue stain) and Western blot analysis of rCD248 protein, including rCD248D1 (6 µg for protein stain and 25 ng for western blot), rCD248D3 (6 µg for protein stain and 140 ng for western blot), and rCD248D1-4 (4.8 µg for protein stain and 40 ng for western blot). **C**, **D** Solid-phase binding assay. **C** Various rCD248 proteins (0.1 µM) were applied to fibronectin (10 µg/mL) - or BSA (as control)-coated multiple−well plate for interaction. The level of interaction was detected with antibodies against the c-myc tag on rCD248 proteins. N = 3. ***P < 0.001. **D** Various fibronectin proteins, including intact fibronectin (10 µg/mL) and equimolar amounts of its various recombinant fragments (70 kDa, 45 kDa, and 30 kDa) were coated onto a multiple-well plate. rCD248D1-4 (0.1 µM) was applied to the well and the interaction was detected by an anti-c-myc antibody. N = 4. *P < 0.05; ***P < 0.001

It has been demonstrated that CD248 interacts with fibronectin. However, the mechanism by which CD248 binds to fibronectin remains unclear. Therefore, rCD248 proteins were prepared (Fig. 3A, B). rCD248D1 (lectin domain) and rCD248D1-4 but not rCD248D3 (EGF-like domain) can bind to fibronectin (Fig. 3C). Moreover, the N-terminal 70 kDa fibronectin was sufficient to bind to the rCD248D1-4 (Fig. 3D). These results suggest that the binding of the lectin domain of CD248 and the N-terminal 70 kDa fragment of fibronectin could mediate melanoma cell-fibronectin interactions.

### rCD248 proteins interfere with melanoma cell adhesion, migration, and VM

**Fig. 4:**
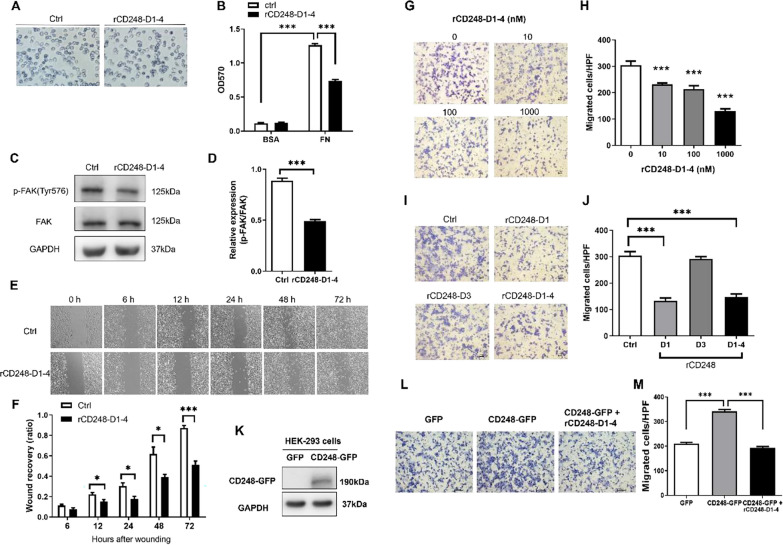
rCD248 interferes with melanoma cell adhesion and migration. **A**, **B** Cell adhesion assay. B16F10 cells were allowed to adhere to BSA- or fibronectin (FN)-coated well with or without rCD248 (1 µM rCD248D1-4) for 30 min and followed by crystal violet stain. **A** Representative images of cell adhesion to fibronectin. **B** Statistical analysis thereof. N = 4. ***P < 0.001. **C**, **D** Western blot analysis of FAK activation (phosphorylation of Tyr576 FAK, p-FAK(Tyr576) in cells after cell adhesion assay. N = 4. ***P < 0.001. B16F10 cells were subjected to (E and F) wound recovery migration assay and **G**–**J** chemotactic migration assay. **E** Representative images of wound recovery assay and statistical analysis thereof (**F**). B16F10 cells were treated with rCD248D1-4 (1 µM) or vehicle control (Ctrl) and photographed at various time points. N = 4. **P < 0.01; ***P < 0.001. **G**, **H** Boyden chamber migration assay of B16F10 cells pretreated with various concentrations of rCD248D1-4 protein 1 h before being applied to the upper well of a Boyden chamber and with the fibroblast-cultured conditioned medium (CM) as chemoattractant. Migrated cells were stained **G** and enumerated **H** 3 h after migration. N = 5. ***P < 0.001 compared with vehicle control. **I**, **J** Boyden chamber migration assay of B16F10 cells pre-treated with 1 µM of various rCD248 proteins (rCD248D1, rCD248D3, and rCD248D1-4) for 1 h before being applied to the upper well of a Boyden chamber and with the CM at the bottom well as chemoattractant. **I** Representative images of Boyden chamber migration assay. Migrated cells were stained and enumerated 3 h after migration. **J** The statistical analysis of Boyden chamber migration assay of B16F10 cells treated with different rCD248 proteins. N = 5. ***P < 0.001 compared with control (Ctrl). HEK293 cells transfected with GFP-tagged CD248 or with GFP as control were subjected to **K** western blot analysis of protein expression and **L**, **M** Boyden chamber migration assay. **L** Representative images of Boyden chamber migration assay in HEK293 cells with and without rCD248D1-4 (1 µM) treatment. Migrated cells were stained and enumerated 4 h after migration. **M** Statistical analysis of Boyden chamber migration assay of HEK293 cells. N = 6. ***P < 0.001

**Fig. 5:**
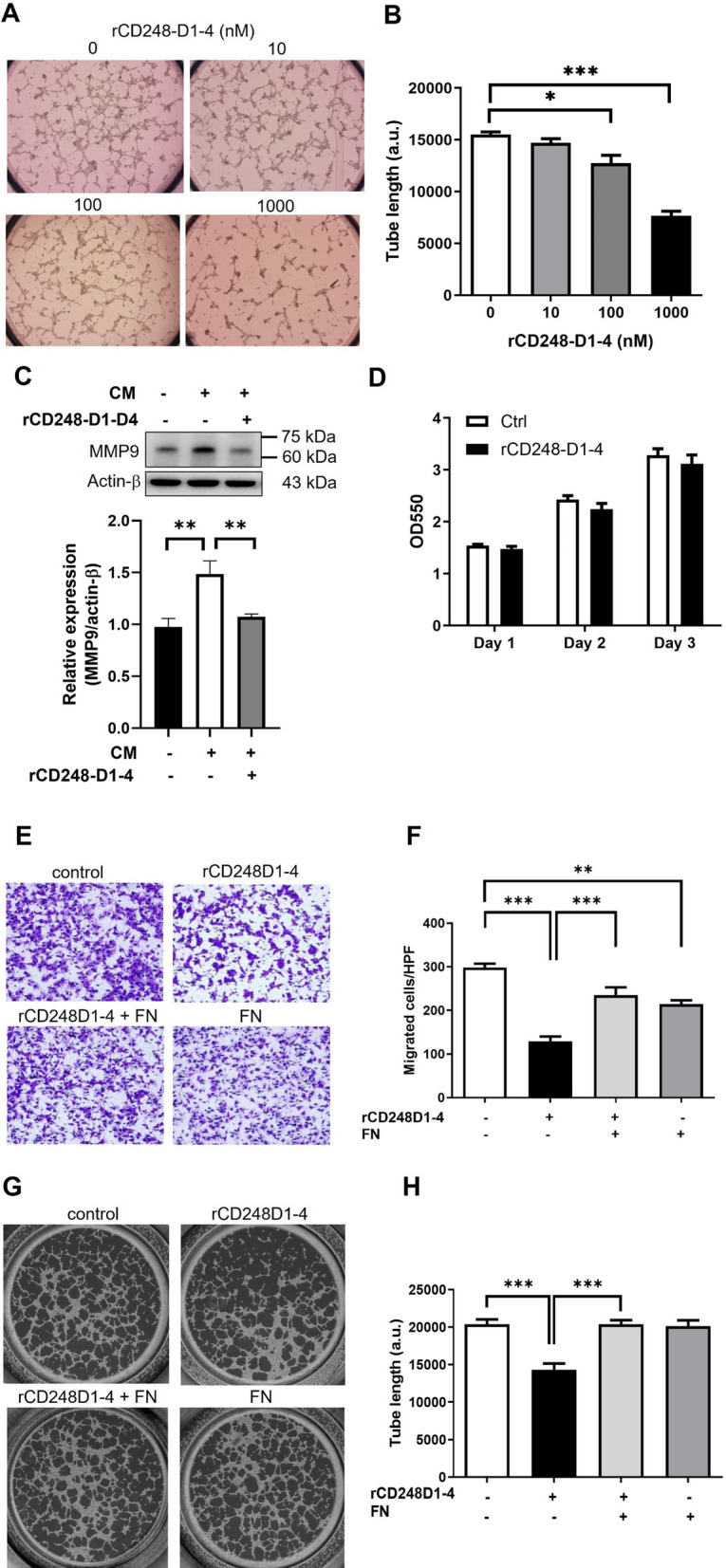
rCD248 interferes with melanoma vascular mimicry by binding to fibronectin. B16F10 cells treated with rCD248 proteins were subjected to **A**, **B** VM assay (network formation on Matrigel), **C** western blot analysis of MMP9 expression, and **D** cell proliferation assay (MTT assay). **A** Representative images of VM assay induced by the fibroblast-cultured conditioned medium (CM). **B** Statistical analysis of the total length of the network was measured 6 h after seeding on Matrigel. N = 3. *P < 0.05; ***P < 0.001 compared with vehicle control. **C** Western blot analysis of MMP9 expression in cells treated with CM in the presence of rCD248D1-4 (1 µM) for 1 day. N = 6. **P < 0.01. **D** B16F10 cells were cultured with or without rCD248D1-D4 for 1 to 3 days and followed by an MTT assay at indicated time point. N = 3. Soluble fibronectin reversed the inhibitory effects of rCD248 proteins on **E**, **F** cell migration and **G**, **H** VM. Soluble fibronectin (0.5 µM) was pre-incubated with rCD248D1-4 (0.5 µM) proteins for 30 min before the indicated experiments. (E) Representative images of the Boyden chamber migration assay using CM as a chemoattractant. **F** Statistical analysis of cell migration toward CM for 3 h. N = 4. **P < 0.01; ***P < 0.001. **G** Representative images of the VM assay. **H** Statistical analysis of the VM assay induced by CM. N = 4. ***P < 0.001

Our results indicated that native CD248 expression in melanoma cells promotes cell adhesion, migration, and VM. Additionally, the lectin domain of CD248 mediated its interaction with fibronectin. Thus, we assumed that exogenous rCD248 proteins can act as a molecular decoy to interfere with cellular functions. The cell adhesion assay showed that fibronectin coating promoted cell adhesion, which was strongly inhibited by rCD248D1-4 (Fig. 4A, B), suggesting that rCD248 may compete for fibronectin binding with melanoma CD248 and impede cell adhesion. This speculation was supported by the reduction in phosphorylated FAK levels (Fig. 4C, D) by rCD248D1-4 during cell adhesion. Dynamic cell adhesion is critical for cell migration. Thus, we further investigated if rCD248D1-4 affects cell migration. Both horizontal cell migration (Fig. 4E, F) and vertical chemotactic migration (Fig. 4G, H) were inhibited by rCD248D1-4 treatment. Interestingly, rCD248D1 but not rCD248D3 exhibited a suppressive effect on chemotactic migration (Fig. 4I, J), suggesting that the lectin domain of CD248 mediates cell migration. We further investigate if rCD248 acts as a molecular decoy using exogenous CD248 expression in HEK293 cells. Western blot analysis showed that GFP-tagged CD248 was successfully expressed in CD248-null HEK293 cells (Fig. 4K). Exogenous CD248 expression promotes cell migration in HEK293 cells when compared with GFP-expressing cells (Fig. 4L, M). The increase of cell migration by CD248 expression was decreased in the presence of rCD248D1-4 protein (Fig. 4L, M), suggesting that rCD248 proteins could function as a molecular decoy to interfere with cell migration. Moreover, we demonstrated that the induction of VM (Fig. 5A, B) and MMP9 protein level (Fig. 5C) by CM were reduced by rCD248D1-4, while cell proliferation was not affected (Fig. 5D). We further addressed whether the suppressive effect of rCD248D1-4 proteins was due to its interaction with fibronectin using the Boyden chamber migration assay and the VM assay. The results showed that the inhibitory effect of rCD248 proteins on the chemotactic migration (Fig. 5E F) and VM activity (Fig. 5G H) of melanoma cells was reversed when rCD248 proteins were pre-incubated with soluble fibronectin. These results suggested that rCD248D1-4 may act as a tumor suppressor at least in part through its interaction with fibronectin.

### rCD248D1-4 inhibits experimental lung metastasis in mice

**Fig. 6:**
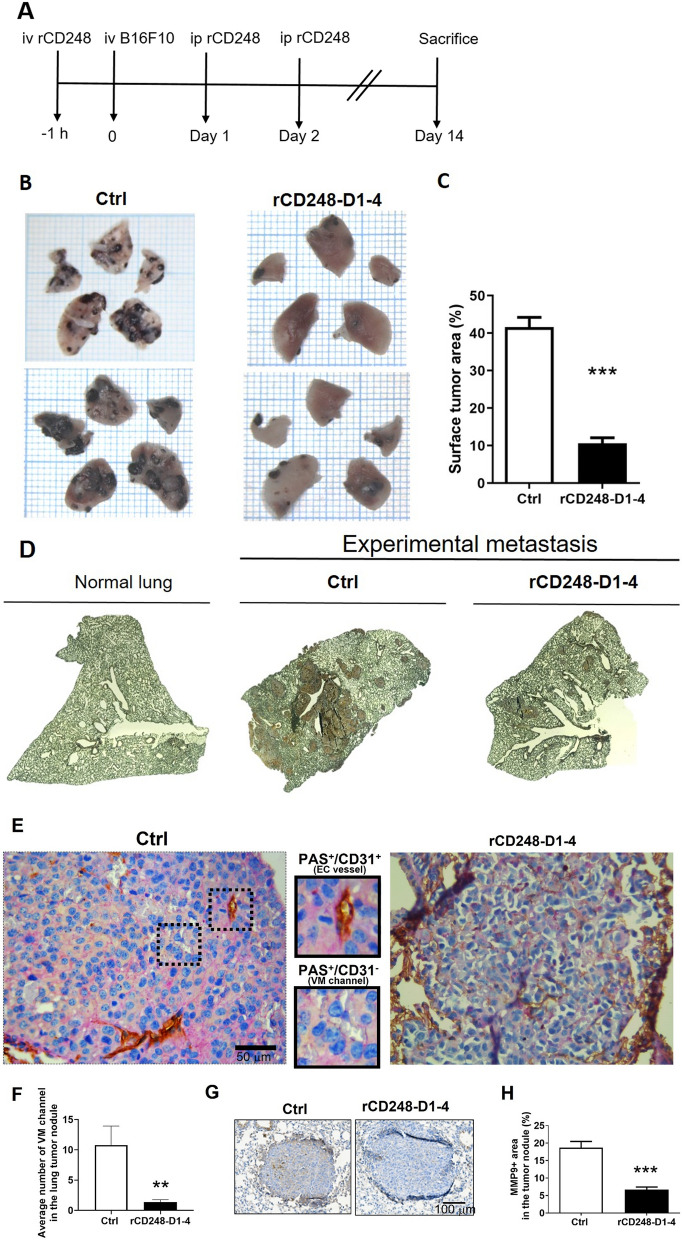
rCD248 inhibits experimental lung metastasis in mice. Experimental lung metastasis assay. **A** The experimental protocol of mouse model of lung metastasis assay. Twenty µg of rCD248D1-4 protein was given 1 h before and 1 and 2 days after intravenous inoculation of B16F10 cells into each mouse. **B** Representative pictures of the gross view of mouse lung surface 14 days after tumor inoculation. **C** Statistical analysis of lung surface occupation of tumor nodules. N = 9 for the control group (Ctrl) and N = 8 for the rCD248D1-4 group. ***, *P* < 0.001. **D** Representative CD248 expression patterns in mouse lung isolated from normal mice and mice after experimental metastasis assay. **E** Representative images of the CD31 (brown color) and PAS (magenta color) double stain in the lung tumor nodules. The PAS^+^/CD31^−^ area is denoted as vascular mimicry (VM) phenotype. **F** Statistical analysis of VM channel (PAS^+^/CD31^−^ stain) in the lung tumor nodule. **P < 0.01. N = 20 nodules for the Ctrl group and N = 21 nodules for the rCD248D1-4 group. **G** Representative images of MMP9 stain in the lung tumor nodules and **H** the statistical analysis thereof. ***P < 0.001. N = 117 nodules for the Ctrl group and N = 69 nodules for the rCD248D1-4 group

To further test our hypothesis that rCD248D1-4 functions as a tumor suppressor, we used an in vivo mouse model of experimental metastasis. The experimental protocol of rCD248D1-4 treatment is shown in Fig. 6A. The mice receiving rCD248D1-4 treatment exhibited less lung surface tumor burden as compared to those receiving vehicle treatment (Fig. 6B, C). Similar results were obtained when micrometastases in the mouse lungs were analyzed (Fig. 6D). Notably, rCD248D1-4 treatment did not alter tumor CD248 expression (Fig. 6D); however, it reduced the expression of PAS^+^/CD31^−^ vessel-like structures (as demonstrated in the insert of Fig. 6E) (Fig. 6E, F) and the expression of MMP9 in the tumor nodules (Fig. 6G, H). These results indicate that rCD248D1-4 could inhibit VM and tumor metastasis.

## Discussion

The occurrence of angiogenesis inside a tumor mass represents a limiting factor for tumor growth over a few centimeters in diameter. Some tumor cells in the tumor mass can evolve to express membrane proteins, assume the characteristics of endothelial cells, and participate in a neovascularization process called VM. Several membrane proteins associated with VM have been identified, including CD31 [29] and VE-cadherin [12]. Stromal-enriched factors have been rarely studied, although they play a critical role in modulating the tumor microenvironment. VM can aggravate aggressive tumor progression and is inversely associated with patient survival rates in several cancer types, such as malignant melanoma and lung cancer. Recent studies have indicated that stromal CD248 expression in fibroblasts and pericytes plays a role in tissue fibrosis and tumor progression [17, 20, 30]. It has been shown that cancer VM phenotype is inversely associated with melanoma patient survival, whereas there is no report on the survival correlation with CD248 expression level in melanoma patients. Hong et al. demonstrated that CD248 expression level is inversely correlated with lung cancer patient survival [30]. CD248 expression has also been observed in some tumor cells [21, 31]. In this study, we found that tumor cells with autonomous CD248 expression exhibited VM expression potential to support blood supply in the tumor and promoted tumor growth and metastasis; therefore, CD248 expression might contribute to tumor malignancy.

We demonstrated that CD248 expression in melanoma tumor cells is correlated with tumor cell-fibronectin interaction, FAK activation, MMP9 expression, cell migration, and VM. The lectin domain at the N-terminal of CD248 may enhance the adherence of tumor cells to fibronectin, whereas its C-terminal cytoplasmic domain may anchor to F-actin, thereby forming a linkage between F-actin and ECM proteins. This linkage could promote cell anchoring to the ECM and cell migration. When the linkage function of CD248 is interrupted by the addition of decoy molecules such as rCD248 proteins in this study, the deletion of its cytoplasmic domain, or the knockdown of CD248 protein expression, cell migration activity is suppressed [25, 32]. Notably, VM is correlated with CD248 expression, indicating that membrane-anchoring proteins are essential for VM formation. Furthermore, melanoma cell adhesion to ECM proteins and cell migration activity can be inhibited by the exogenous addition of rCD248 proteins; VM on Matrigel and VM pattern in experimental lung metastasis mouse model were also inhibited by rCD248 proteins. Furthermore, the rCD248 protein suppressed tumor metastasis in mice. These results indicate that CD248 expression is associated with an aggressive phenotypic switch of melanoma, having better metastatic activity and VM function, partly by forming a linkage between tumor cells and ECM protein fibronectin.

It is still unclear how CD248 expression causes cells to assume VM function, although the lectin domain of CD 248 is essential for its function. In this study, we found that the lectin domain of CD248, not the EGF-like domain, was required for its interaction with fibronectin. Cell adhesion and FAK activity were compromised in cells with CD248-knockdown or rCD248D1-4 treatment during cell adhesion to fibronectin. Furthermore, we demonstrated that inhibition of cell migration by rCD248 was associated with its lectin domain but not the EGF-like domain. These results imply that the lectin domain of CD248 may be involved in melanoma cell–ECM interactions and cell migration. Though the current results did not directly test whether rCD248 suppresses tumor lung metastasis through interaction with fibronectin, our results, including protein-based and cell-based interaction assays and in vitro assays, all suggest that rCD248 could interfere with membrane-bound CD248 in melanoma migration and VM in part through the interference of cell-ECM interaction.

FAK signaling and MMPs have been associated with tumor VM [13]. In line with these observations, we demonstrated that CD248 expression modulates cell-ECM adhesion, FAK activation, and MMP9 expression in melanoma cells. In addition, exogenous expression of CD248 in the CD248-null HEK293 cells promotes cell migration, which was reduced in the presence of the rCD248 protein. These results suggest that CD248 is a molecule that can promote dynamic cell activity such as cell migration and VM, thereby transforming malignant melanoma tumors. Though we have demonstrated that CD248 expression level was associated with several cellular activities, whether CD248-mediated VM is responsible for tumor progression has not been directly tested in the current study. To address this question, constitutive activation of FAK signaling to overcome FAK suppression induced by knockdown of CD248 or rCD248 treatment in melanoma can be utilized.

The functions of CD248 in tumors might not be limited to the cell-matrix interaction as its expression level has been correlated with the activities of the cell in different cell types, including the activity of cell adhesion to the matrix, migration, proliferation, and modulation of signal transduction. For instance, we demonstrated that CD248 expression plays a role in activated myofibroblast proliferation and migration and modulates PDGF receptor signaling [25]. Consequently, CD248 expression in myofibroblasts is closely associated with cutaneous wound healing [25]. CD248 in pericytes regulates cell proliferation and modulates pericyte–endothelial cell interaction [33, 34]. In contrast, CD248 expression in CD8^+^ T cells suppressed cell proliferation [35]. It is unclear whether CD248 has a proliferative effect on macrophages. Our recent study demonstrated that macrophages with lectin domain-deleted CD248 exhibit less pro-inflammatory reactions induced by LPS, and mice with this genetic background respond less to LPS-induced septic shock [36], suggesting that CD248 has the potential to regulate toll-like receptor signaling similar to thrombomodulin [37]. Here, we demonstrated that CD248 in melanoma cells also plays an important role in cell adhesion, migration, and VM. However, CD248 expression had less of an impact on melanoma cell proliferation. Based on these observations, we can conclude that CD248 plays a critical role in cell-matrix adhesion and cell migration in most studied cell types. However, the expression of CD248 may not have similar effects on cell proliferation in different cell types. Since CD248 could act as a co-receptor to modulate membrane receptor activity, as demonstrated in regulating PDGF signaling [25, 33, 38, 39], the cell-type specific effects of CD248 on cell proliferation may be attributed to the nature of different membrane receptors for growth factors existing in different cell types.

CD248 has been proposed as a valuable biomarker for evaluating tumor progression in renal cell carcinoma, soft tissue sarcoma, glioblastoma, colorectal cancer, bladder cancer, and melanoma [23, 40–43]. CD248 promotes tumor progression through both tumor cell-autonomous and non-cell-autonomous effects. Through a non-autonomous effect, stromal CD248 can promote tumor progression [32] by modulating the tumor microenvironment. For example, CD248 expressed in tumor-associated pericytes facilitates distal dissemination in a contact-dependent manner, thereby increasing circulating tumor cell numbers [20]. Moreover, CD248 regulates Wnt signaling in pericytes to promote angiogenesis and tumor growth in lung cancer [30]. CD248 expression in cancer-associated fibroblasts promotes hepatocellular carcinoma progression through interaction with CD68 on macrophages and consequent polarization to the tumor-promoting M2 phenotype [44]. In contrast, CD248 expression in some cancer cells may contribute to tumor progression through autonomous effects. For example, CD248 expression in osteosarcoma may contribute to cancer invasion and metastasis [21]. Our results show that melanoma CD248 contributes to cell migration and VM in part through modulating cell-ECM adhesion and MMP9 expression, which could promote tumor metastasis. These studies indicate that CD248 may be a promising therapeutic target for cancer treatment.

Owing to its oncofetal gene-like expression pattern, targeting CD248 has been suggested for treating several disorders, such as tissue fibrosis and cancer [17, 45]. Either by drug conjugation or by inducing internalization and degradation, antibodies against CD248 have been intensively developed [46]. Recent studies indicate that humanized monoclonal antibodies against CD248 show a significant pre-clinical effect on the suppression of tumor progression partly through the reduction of membrane CD248 expression [47] and maybe because of the inhibition of cell-ECM interaction [16]; however, the beneficial effects on cancer patients with CD248 antibody treatment was limited [48, 49]. CD248 may have multiple ECM-binding sites because it has various interacting partners, which has been demonstrated in several studies [16, 25, 44, 50, 51]. Therefore, the good and bad of being a monovalent monoclonal antibody can only block one site, which limits the use of the existing CD248 antibody. In this regard, molecular mimics and receptor decoys are promising alternatives for treating various disorders. Accordingly, molecular decoys that can fine-tune endogenous protein functions have been proposed as novel therapeutics for fighting cancer and inflammatory disease [52]. Treatment with rCD248 leads to a pronounced reduction in tumor in vitro and in vivo, suggesting that decoy molecules, such as rCD248D1-4, may have the potential to interfere with tumor progression. Although we have identified that the lectin domain of CD248 could be the functional region of CD248 that interferes with melanoma VM and metastasis, more specific sequences/regions in the lectin domain might be clearly defined in the future. Thus, synthetic peptides of active CD248 molecular decoys hold promise for treating cancer and fibrotic diseases.

## Conclusion

In summary, our study demonstrated that melanoma CD248 promotes tumor metastatic behavior by enhancing cell-fibronectin interaction, migration, and VM activity. The lectin domain of CD248 and the N-terminal 70 kDa fragment of fibronectin mediate molecular interactions and may be responsible for subsequent VM and metastasis. Using rCD248, we demonstrated that the cell adhesion to fibronectin, migration, and VM in vitro and lung metastasis of melanoma in mice, were distinctly reduced. Thus, we conclude that melanoma CD248 promotes the malignant transformation of melanoma and could be a therapeutic target for cancer.

## Data Availability

The data included and materials used in this study are available from the corresponding author upon reasonable request.

## Abbreviations

VM: Vascular mimicry
EGF: Epidermal growth factor
CM: Conditioned medium
FAK: Focal adhesion kinase
rCD248: Recombinant CD248
PAS: Periodic acid-Schiff
siCtrl: Scrambled siRNA control
siCd248: CD248 siRNA
PDGFR: Platelet-derived growth factor receptor
ECM: Extracellular matrix
