# Correction: Interference in melanoma CD248 function reduces vascular mimicry and metastasis

**DOI:** 10.1186/s12929-025-01155-5

**Published:** 2025-07-11

**Authors:** Cheng-Hsiang Kuo, Ya-Fang Wu, Bi-Ing Chang, Chao-Kai Hsu, Chao-Han Lai, Hua-Lin Wu

**Affiliations:** 1 International Center for Wound Repair and Regeneration, National Cheng Kung University, Tainan, Taiwan; 2 Department of Biochemistry and Molecular Biology, College of Medicine, National Cheng Kung University, No. 1, University Road, 701 Tainan, Taiwan; 3 Department of Dermatology, National Cheng Kung University Hospital, College of Medicine, National Cheng Kung University, Tainan, Taiwan; 4 Department of Surgery, National Cheng Kung University Hospital, College of Medicine, National Cheng Kung University, Tainan, Taiwan

**Correction to: Journal of Biomedical Science (2022) 29:98** 10.1186/s12929-022-00882-3

After the article [1] was published, the authors identified errors in Fig. 4 that occurred unintentionally during figure assembly. Specifically, two panels—Panel A and Panel G—contained mistakes and require correction.

Updated Fig. 4 is provided in this article. Importantly, these revisions do not impact on the conclusions or interpretations of the study in any way.

**Fig. 4:**
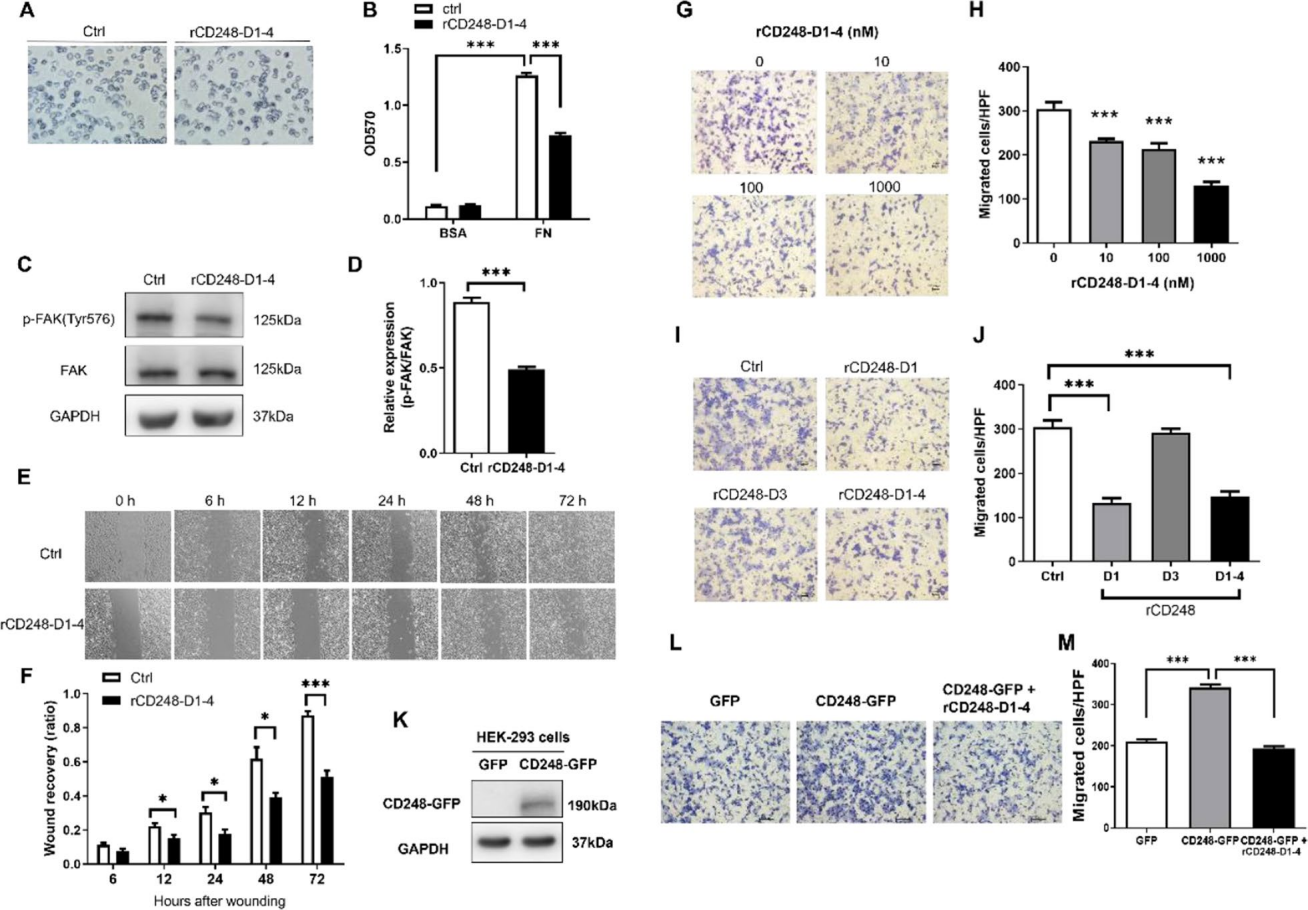
rCD248 interferes with melanoma cell adhesion and migration. **A**, **B** Cell adhesion assay. B16F10 cells were allowed to adhere to BSA- or fibronectin (FN)-coated well with or without rCD248 (1 µM rCD248D1-4) for 30 min and followed by crystal violet stain. **A** Representative images of cell adhesion to fibronectin. **B** Statistical analysis thereof. N = 4. ***P < 0.001. **C**, **D** Western blot analysis of FAK activation (phosphorylation of Tyr576 FAK, p-FAK(Tyr576) in cells after cell adhesion assay. N = 4. ***P < 0.001. B16F10 cells were subjected to (E and F) wound recovery migration assay and **G**–**J** chemotactic migration assay. **E** Representative images of wound recovery assay and statistical analysis thereof (**F**). B16F10 cells were treated with rCD248D1-4 (1 µM) or vehicle control (Ctrl) and photographed at various time points. N = 4. **P < 0.01; ***P < 0.001. **G**, **H** Boyden chamber migration assay of B16F10 cells pretreated with various concentrations of rCD248D1-4 protein 1 h before being applied to the upper well of a Boyden chamber and with the fibroblast-cultured conditioned medium (CM) as chemoattractant. Migrated cells were stained **G** and enumerated **H** 3 h after migration. N = 5. ***P < 0.001 compared with vehicle control. **I**, **J** Boyden chamber migration assay of B16F10 cells pre-treated with 1 µM of various rCD248 proteins (rCD248D1, rCD248D3, and rCD248D1-4) for 1 h before being applied to the upper well of a Boyden chamber and with the CM at the bottom well as chemoattractant. **I** Representative images of Boyden chamber migration assay. Migrated cells were stained and enumerated 3 h after migration. **J** The statistical analysis of Boyden chamber migration assay of B16F10 cells treated with different rCD248 proteins. N = 5. ***P < 0.001 compared with control (Ctrl). HEK293 cells transfected with GFP-tagged CD248 or with GFP as control were subjected to **K** western blot analysis of protein expression and **L**, **M** Boyden chamber migration assay. **L** Representative images of Boyden chamber migration assay in HEK293 cells with and without rCD248D1-4 (1 µM) treatment. Migrated cells were stained and enumerated 4 h after migration. **M** Statistical analysis of Boyden chamber migration assay of HEK293 cells. N = 6. ***P < 0.001

The authors sincerely regret this inadvertent error and apologize for any confusion it may have caused.

The original paper has been updated.
